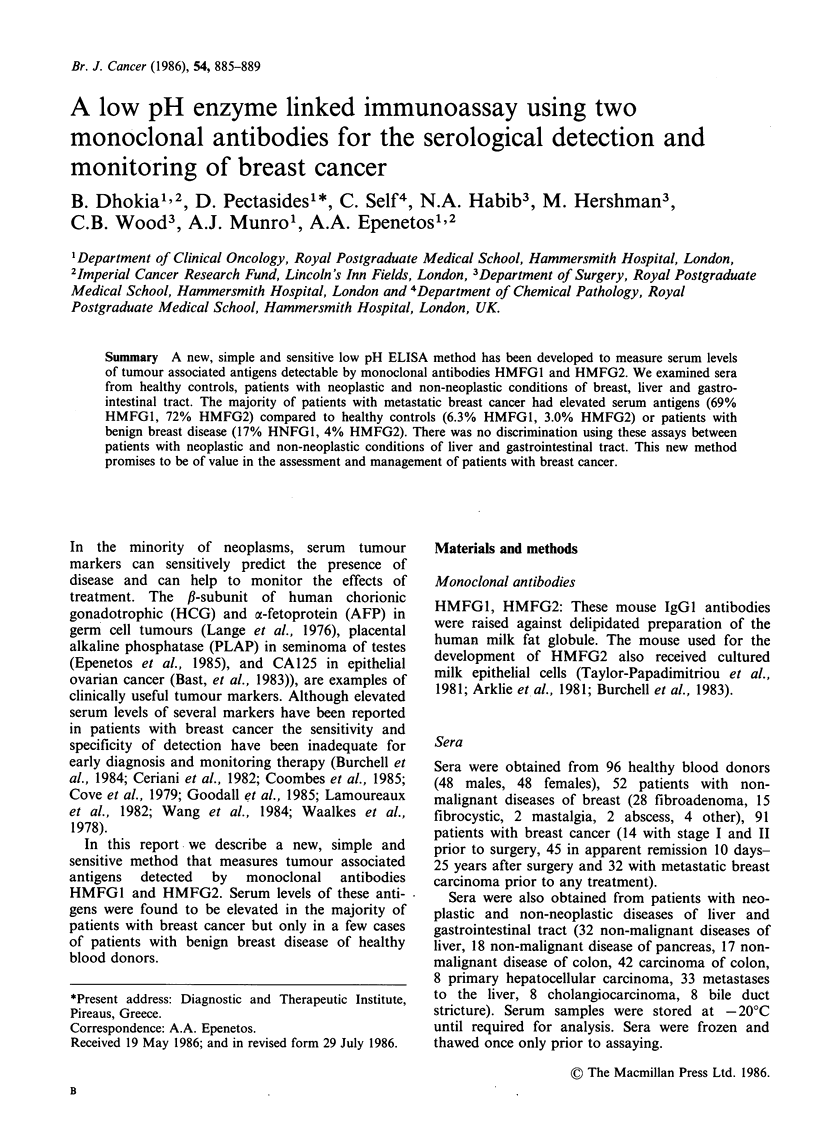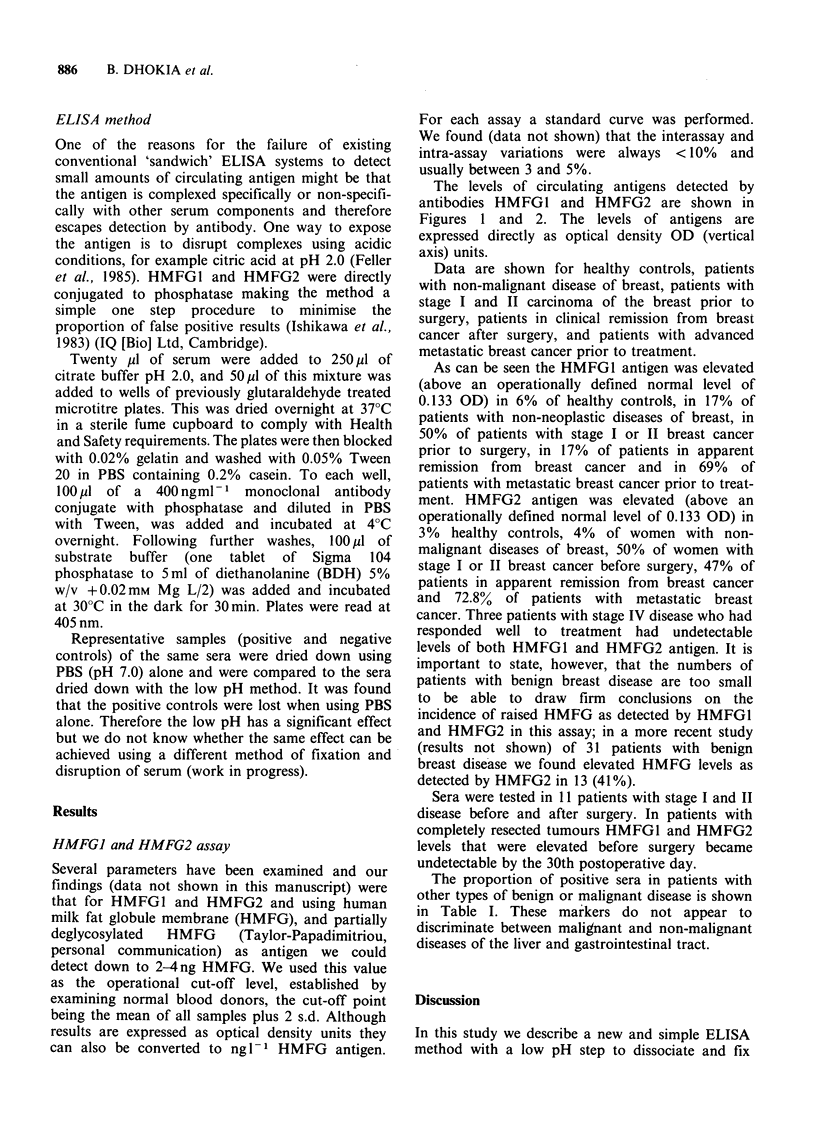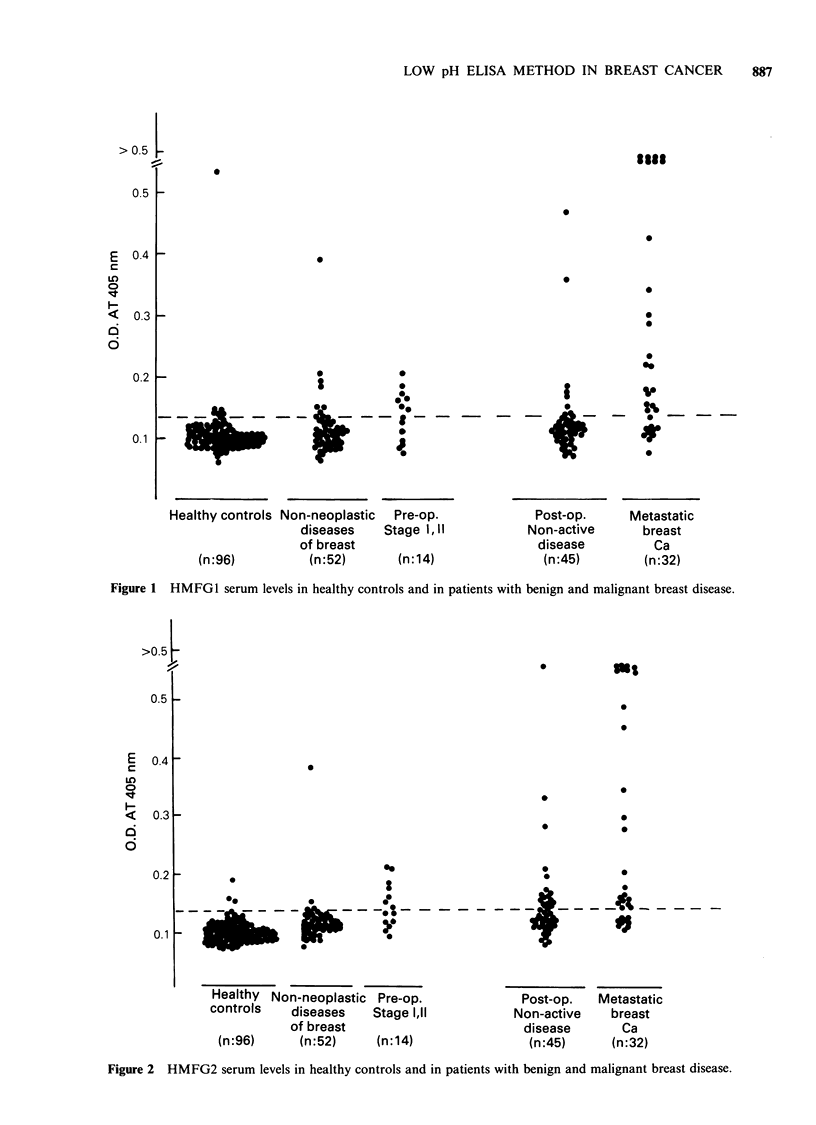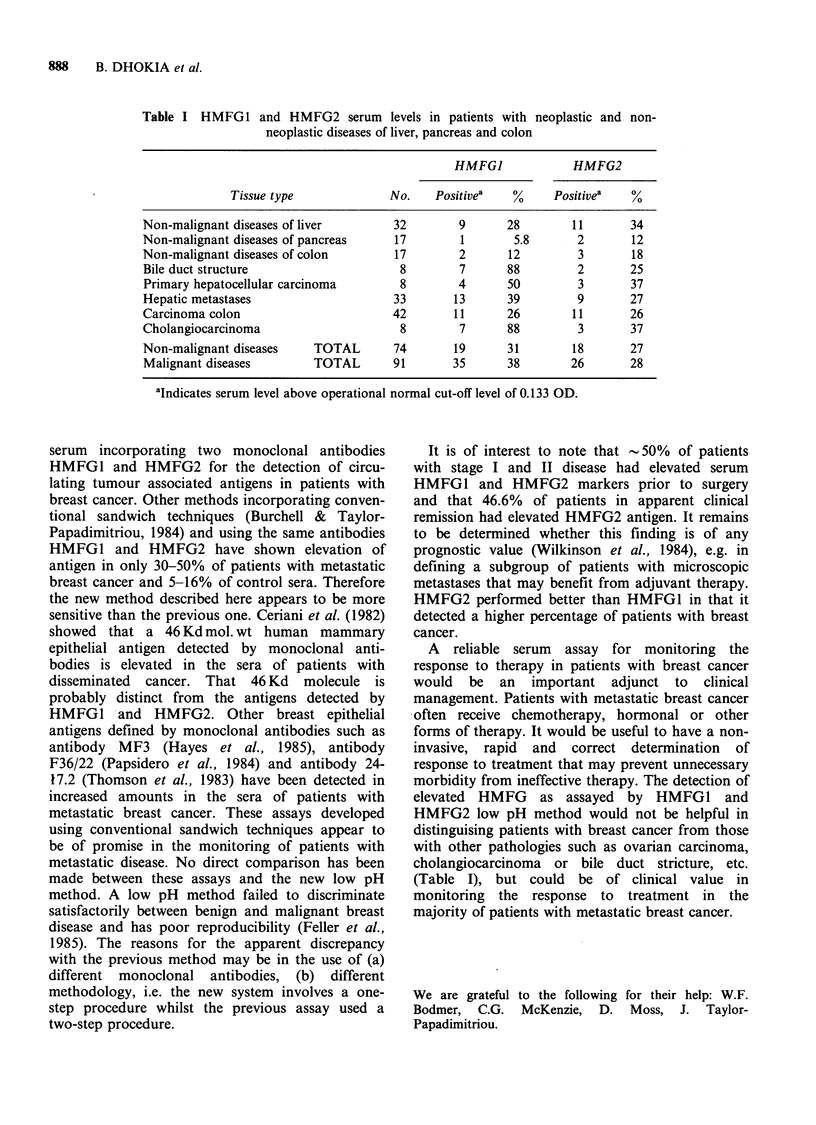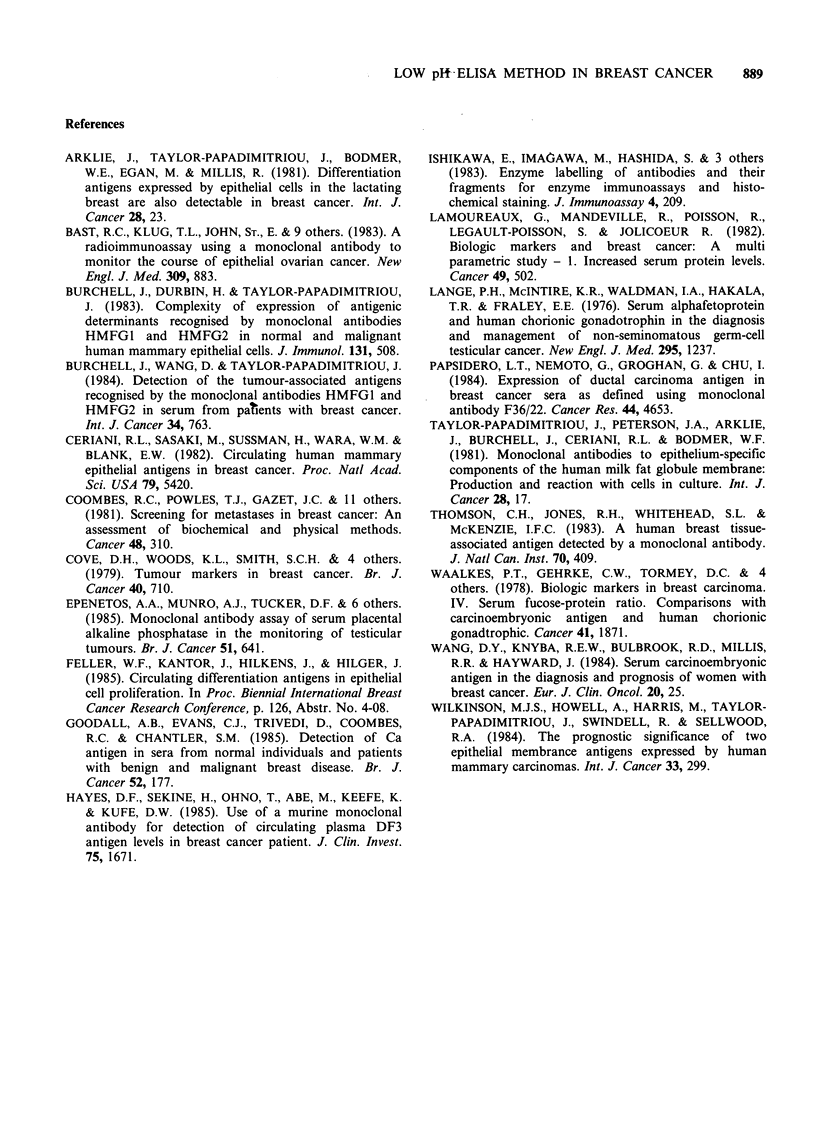# A low pH enzyme linked immunoassay using two monoclonal antibodies for the serological detection and monitoring of breast cancer.

**DOI:** 10.1038/bjc.1986.257

**Published:** 1986-12

**Authors:** B. Dhokia, D. Pectasides, C. Self, N. A. Habib, M. Hershman, C. B. Wood, A. J. Munro, A. A. Epenetos

## Abstract

A new, simple and sensitive low pH ELISA method has been developed to measure serum levels of tumour associated antigens detectable by monoclonal antibodies HMFG1 and HMFG2. We examined sera from healthy controls, patients with neoplastic and non-neoplastic conditions of breast, liver and gastrointestinal tract. The majority of patients with metastatic breast cancer had elevated serum antigens (69% HMFG1, 72% HMFG2) compared to healthy controls (6.3% HMFG1, 3.0% HMFG2) or patients with benign breast disease (17% HMFG1, 4% HMFG2). There was no discrimination using these assays between patients with neoplastic and non-neoplastic conditions of liver and gastrointestinal tract. This new method promises to be of value in the assessment and management of patients with breast cancer.


*Br. J. Cancer* (1986), **54**, 885–889

### A low pH enzyme linked immunoassay using two monoclonal antibodies for the serological detection and monitoring of breast cancer

B. Dhokia[1,2], D. Pectasides[1]*, C. Self[4], N.A. Habib[3], M. Hershman[3], C.B. Wood[3], A.J. Munro[1], A.A. Epenetos[1,2]

[1]*Department of Clinical Oncology, Royal Postgraduate Medical School, Hammersmith Hospital, London,* [2]*Imperial Cancer Research Fund, Lincoln's Inn Fields, London,* [3]*Department of Surgery, Royal Postgraduate Medical School, Hammersmith Hospital, London and* [4]*Department of Chemical Pathology, Royal Postgraduate Medical School, Hammersmith Hospital, London, UK.*

**Summary** A new, simple and sensitive low pH ELISA method has been developed to measure serum levels of tumour associated antigens detectable by monoclonal antibodies HMFG1 and HMFG2. We examined sera from healthy controls, patients with neoplastic and non-neoplastic conditions of breast, liver and gastrointestinal tract. The majority of patients with metastatic breast cancer had elevated serum antigens (69% HMFG1, 72% HMFG2) compared to healthy controls (6.3% HMFG1, 3.0% HMFG2) or patients with benign breast disease (17% HNFG1, 4% HMFG2). There was no discrimination using these assays between patients with neoplastic and non-neoplastic conditions of liver and gastrointestinal tract. This new method promises to be of value in the assessment and management of patients with breast cancer.

In the minority of neoplasms, serum tumour markers can sensitively predict the presence of disease and can help to monitor the effects of treatment. The $\beta$-subunit of human chorionic gonadotrophic (HCG) and $\alpha$-fetoprotein (AFP) in germ cell tumours (Lange *et al.*, 1976), placental alkaline phosphatase (PLAP) in seminoma of testes (Epenetos *et al.*, 1985), and CA125 in epithelial ovarian cancer (Bast, *et al.*, 1983)), are examples of clinically useful tumour markers. Although elevated serum levels of several markers have been reported in patients with breast cancer the sensitivity and specificity of detection have been inadequate for early diagnosis and monitoring therapy (Burchell *et al.*, 1984; Ceriani *et al.*, 1982; Coombes *et al.*, 1985; Cove *et al.*, 1979; Goodall *et al.*, 1985; Lamoureaux *et al.*, 1982; Wang *et al.*, 1984; Waalkes *et al.*, 1978).

In this report we describe a new, simple and sensitive method that measures tumour associated antigens detected by monoclonal antibodies HMFG1 and HMFG2. Serum levels of these antigens were found to be elevated in the majority of patients with breast cancer but only in a few cases of patients with benign breast disease of healthy blood donors.

*Present address: Diagnostic and Therapeutic Institute, Pireaus, Greece.

Correspondence: A.A. Epenetos.

Received 19 May 1986; and in revised form 29 July 1986.

### Materials and methods

### *Monoclonal antibodies*

HMFG1, HMFG2: These mouse IgG1 antibodies were raised against delipidated preparation of the human milk fat globule. The mouse used for the development of HMFG2 also received cultured milk epithelial cells (Taylor-Papadimitriou *et al.*, 1981; Arklie *et al.*, 1981; Burchell *et al.*, 1983).

### *Sera*

Sera were obtained from 96 healthy blood donors (48 males, 48 females), 52 patients with non-malignant diseases of breast (28 fibroadenoma, 15 fibrocystic, 2 mastalgia, 2 abscess, 4 other), 91 patients with breast cancer (14 with stage I and II prior to surgery, 45 in apparent remission 10 days–25 years after surgery and 32 with metastatic breast carcinoma prior to any treatment).

Sera were also obtained from patients with neoplastic and non-neoplastic diseases of liver and gastrointestinal tract (32 non-malignant diseases of liver, 18 non-malignant disease of pancreas, 17 non-malignant disease of colon, 42 carcinoma of colon, 8 primary hepatocellular carcinoma, 33 metastases to the liver, 8 cholangiocarcinoma, 8 bile duct stricture). Serum samples were stored at $-20°C$ until required for analysis. Sera were frozen and thawed once only prior to assaying.

© The Macmillan Press Ltd. 1986.

*ELISA method*

One of the reasons for the failure of existing conventional 'sandwich' ELISA systems to detect small amounts of circulating antigen might be that the antigen is complexed specifically or non-specifically with other serum components and therefore escapes detection by antibody. One way to expose the antigen is to disrupt complexes using acidic conditions, for example citric acid at pH 2.0 (Feller *et al.*, 1985). HMFG1 and HMFG2 were directly conjugated to phosphatase making the method a simple one step procedure to minimise the proportion of false positive results (Ishikawa *et al.*, 1983) (IQ [Bio] Ltd, Cambridge).

Twenty $\mu$l of serum were added to 250 $\mu$l of citrate buffer pH 2.0, and 50 $\mu$l of this mixture was added to wells of previously glutaraldehyde treated microtitre plates. This was dried overnight at 37°C in a sterile fume cupboard to comply with Health and Safety requirements. The plates were then blocked with 0.02% gelatin and washed with 0.05% Tween 20 in PBS containing 0.2% casein. To each well, 100 $\mu$l of a 400 $ng\,ml^{-1}$ monoclonal antibody conjugate with phosphatase and diluted in PBS with Tween, was added and incubated at 4°C overnight. Following further washes, 100 $\mu$l of substrate buffer (one tablet of Sigma 104 phosphatase to 5 ml of diethanolanine (BDH) 5% w/v +0.02 mM Mg L/2) was added and incubated at 30°C in the dark for 30 min. Plates were read at 405 nm.

Representative samples (positive and negative controls) of the same sera were dried down using PBS (pH 7.0) alone and were compared to the sera dried down with the low pH method. It was found that the positive controls were lost when using PBS alone. Therefore the low pH has a significant effect but we do not know whether the same effect can be achieved using a different method of fixation and disruption of serum (work in progress).

### Results

*HMFG1 and HMFG2 assay*

Several parameters have been examined and our findings (data not shown in this manuscript) were that for HMFG1 and HMFG2 and using human milk fat globule membrane (HMFG), and partially deglycosylated HMFG (Taylor-Papadimitriou, personal communication) as antigen we could detect down to 2–4 ng HMFG. We used this value as the operational cut-off level, established by examining normal blood donors, the cut-off point being the mean of all samples plus 2 s.d. Although results are expressed as optical density units they can also be converted to $ng\,l^{-1}$ HMFG antigen. For each assay a standard curve was performed. We found (data not shown) that the interassay and intra-assay variations were always <10% and usually between 3 and 5%.

The levels of circulating antigens detected by antibodies HMFG1 and HMFG2 are shown in Figures 1 and 2. The levels of antigens are expressed directly as optical density OD (vertical axis) units.

Data are shown for healthy controls, patients with non-malignant disease of breast, patients with stage I and II carcinoma of the breast prior to surgery, patients in clinical remission from breast cancer after surgery, and patients with advanced metastatic breast cancer prior to treatment.

As can be seen the HMFG1 antigen was elevated (above an operationally defined normal level of 0.133 OD) in 6% of healthy controls, in 17% of patients with non-neoplastic diseases of breast, in 50% of patients with stage I or II breast cancer prior to surgery, in 17% of patients in apparent remission from breast cancer and in 69% of patients with metastatic breast cancer prior to treatment. HMFG2 antigen was elevated (above an operationally defined normal level of 0.133 OD) in 3% healthy controls, 4% of women with non-malignant diseases of breast, 50% of women with stage I or II breast cancer before surgery, 47% of patients in apparent remission from breast cancer and 72.8% of patients with metastatic breast cancer. Three patients with stage IV disease who had responded well to treatment had undetectable levels of both HMFG1 and HMFG2 antigen. It is important to state, however, that the numbers of patients with benign breast disease are too small to be able to draw firm conclusions on the incidence of raised HMFG as detected by HMFG1 and HMFG2 in this assay; in a more recent study (results not shown) of 31 patients with benign breast disease we found elevated HMFG levels as detected by HMFG2 in 13 (41%).

Sera were tested in 11 patients with stage I and II disease before and after surgery. In patients with completely resected tumours HMFG1 and HMFG2 levels that were elevated before surgery became undetectable by the 30th postoperative day.

The proportion of positive sera in patients with other types of benign or malignant disease is shown in Table I. These markers do not appear to discriminate between malignant and non-malignant diseases of the liver and gastrointestinal tract.

### Discussion

In this study we describe a new and simple ELISA method with a low pH step to dissociate and fix

**Figure 1** HMFG1 serum levels in healthy controls and in patients with benign and malignant breast disease.

**Figure 2** HMFG2 serum levels in healthy controls and in patients with benign and malignant breast disease.

**Table I** HMFG1 and HMFG2 serum levels in patients with neoplastic and non-neoplastic diseases of liver, pancreas and colon

| *Tissue type* | | *No.* | *HMFG1* | | *HMFG2* | |
|---|---|---|---|---|---|---|
| | | | *Positive*[a] | % | *Positive*[a] | % |
| Non-malignant diseases of liver | | 32 | 9 | 28 | 11 | 34 |
| Non-malignant diseases of pancreas | | 17 | 1 | 5.8 | 2 | 12 |
| Non-malignant diseases of colon | | 17 | 2 | 12 | 3 | 18 |
| Bile duct structure | | 8 | 7 | 88 | 2 | 25 |
| Primary hepatocellular carcinoma | | 8 | 4 | 50 | 3 | 37 |
| Hepatic metastases | | 33 | 13 | 39 | 9 | 27 |
| Carcinoma colon | | 42 | 11 | 26 | 11 | 26 |
| Cholangiocarcinoma | | 8 | 7 | 88 | 3 | 37 |
| Non-malignant diseases | TOTAL | 74 | 19 | 31 | 18 | 27 |
| Malignant diseases | TOTAL | 91 | 35 | 38 | 26 | 28 |

[a]Indicates serum level above operational normal cut-off level of 0.133 OD.

serum incorporating two monoclonal antibodies HMFG1 and HMFG2 for the detection of circulating tumour associated antigens in patients with breast cancer. Other methods incorporating conventional sandwich techniques (Burchell & Taylor-Papadimitriou, 1984) and using the same antibodies HMFG1 and HMFG2 have shown elevation of antigen in only 30–50% of patients with metastatic breast cancer and 5–16% of control sera. Therefore the new method described here appears to be more sensitive than the previous one. Ceriani *et al.* (1982) showed that a 46 Kd mol. wt human mammary epithelial antigen detected by monoclonal antibodies is elevated in the sera of patients with disseminated cancer. That 46 Kd molecule is probably distinct from the antigens detected by HMFG1 and HMFG2. Other breast epithelial antigens defined by monoclonal antibodies such as antibody MF3 (Hayes *et al.*, 1985), antibody F36/22 (Papsidero *et al.*, 1984) and antibody 24-17.2 (Thomson *et al.*, 1983) have been detected in increased amounts in the sera of patients with metastatic breast cancer. These assays developed using conventional sandwich techniques appear to be of promise in the monitoring of patients with metastatic disease. No direct comparison has been made between these assays and the new low pH method. A low pH method failed to discriminate satisfactorily between benign and malignant breast disease and has poor reproducibility (Feller *et al.*, 1985). The reasons for the apparent discrepancy with the previous method may be in the use of (a) different monoclonal antibodies, (b) different methodology, i.e. the new system involves a one-step procedure whilst the previous assay used a two-step procedure.

It is of interest to note that ~50% of patients with stage I and II disease had elevated serum HMFG1 and HMFG2 markers prior to surgery and that 46.6% of patients in apparent clinical remission had elevated HMFG2 antigen. It remains to be determined whether this finding is of any prognostic value (Wilkinson *et al.*, 1984), e.g. in defining a subgroup of patients with microscopic metastases that may benefit from adjuvant therapy. HMFG2 performed better than HMFG1 in that it detected a higher percentage of patients with breast cancer.

A reliable serum assay for monitoring the response to therapy in patients with breast cancer would be an important adjunct to clinical management. Patients with metastatic breast cancer often receive chemotherapy, hormonal or other forms of therapy. It would be useful to have a non-invasive, rapid and correct determination of response to treatment that may prevent unnecessary morbidity from ineffective therapy. The detection of elevated HMFG as assayed by HMFG1 and HMFG2 low pH method would not be helpful in distinguising patients with breast cancer from those with other pathologies such as ovarian carcinoma, cholangiocarcinoma or bile duct stricture, etc. (Table I), but could be of clinical value in monitoring the response to treatment in the majority of patients with metastatic breast cancer.

We are grateful to the following for their help: W.F. Bodmer, C.G. McKenzie, D. Moss, J. Taylor-Papadimitriou.

### References

ARKLIE, J., TAYLOR-PAPADIMITRIOU, J., BODMER, W.E., EGAN, M. & MILLIS, R. (1981). Differentiation antigens expressed by epithelial cells in the lactating breast are also detectable in breast cancer. *Int. J. Cancer* **28,** 23.

BAST, R.C., KLUG, T.L., JOHN, ST., E. & 9 others. (1983). A radioimmunoassay using a monoclonal antibody to monitor the course of epithelial ovarian cancer. *New Engl. J. Med.* **309,** 883.

BURCHELL, J., DURBIN, H. & TAYLOR-PAPADIMITRIOU, J. (1983). Complexity of expression of antigenic determinants recognised by monoclonal antibodies HMFGl and HMFG2 in normal and malignant human mammary epithelial cells. *J. Immunol.* **131,** 508.

BURCHELL, J., WANG, D. & TAYLOR-PAPADIMITRIOU, J. (1984). Detection of the tumour-associated antigens recognised by the monoclonal antibodies HMFG1 and HMFG2 in serum from patients with breast cancer. *Int. J. Cancer* **34,** 763.

CERIANI, R.L., SASAKI, M., SUSSMAN, H., WARA, W.M. & BLANK, E.W. (1982). Circulating human mammary epithelial antigens in breast cancer. *Proc. Natl Acad. Sci. USA* **79,** 5420.

COOMBES, R.C., POWLES, T.J., GAZET, J.C. & 11 others. (1981). Screening for metastases in breast cancer: An assessment of biochemical and physical methods. *Cancer* **48,** 310.

COVE, D.H., WOODS, K.L., SMITH, S.C.H. & 4 others. (1979). Tumour markers in breast cancer. *Br. J. Cancer* **40,** 710.

EPENETOS, A.A., MUNRO, A.J., TUCKER, D.F. & 6 others. (1985). Monoclonal antibody assay of serum placental alkaline phosphatase in the monitoring of testicular tumours. *Br. J. Cancer* **51,** 641.

FELLER, W.F., KANTOR, J., HILKENS, J., & HILGER, J. (1985). Circulating differentiation antigens in epithelial cell proliferation. In *Proc. Biennial International Breast Cancer Research Conference,* p. 126, Abstr. No. 4-08.

GOODALL, A.B., EVANS, C.J., TRIVEDI, D., COOMBES, R.C. & CHANTLER, S.M. (1985). Detection of Ca antigen in sera from normal individuals and patients with benign and malignant breast disease. *Br. J. Cancer* **52,** 177.

HAYES, D.F., SEKINE, H., OHNO, T., ABE, M., KEEFE, K. & KUFE, D.W. (1985). Use of a murine monoclonal antibody for detection of circulating plasma DF3 antigen levels in breast cancer patient. *J. Clin. Invest.* **75,** 1671.

ISHIKAWA, E., IMAGAWA, M., HASHIDA, S. & 3 others (1983). Enzyme labelling of antibodies and their fragments for enzyme immunoassays and histochemical staining. *J. Immunoassay* **4,** 209.

LAMOUREAUX, G., MANDEVILLE, R., POISSON, R., LEGAULT-POISSON, S. & JOLICOEUR R. (1982). Biologic markers and breast cancer: A multi parametric study – 1. Increased serum protein levels. *Cancer* **49,** 502.

LANGE, P.H., McINTIRE, K.R., WALDMAN, I.A., HAKALA, T.R. & FRALEY, E.E. (1976). Serum alphafetoprotein and human chorionic gonadotrophin in the diagnosis and management of non-seminomatous germ-cell testicular cancer. *New Engl. J. Med.* **295,** 1237.

PAPSIDERO, L.T., NEMOTO, G., GROGHAN, G. & CHU, I. (1984). Expression of ductal carcinoma antigen in breast cancer sera as defined using monoclonal antibody F36/22. *Cancer Res.* **44,** 4653.

TAYLOR-PAPADIMITRIOU, J., PETERSON, J.A., ARKLIE, J., BURCHELL, J., CERIANI, R.L. & BODMER, W.F. (1981). Monoclonal antibodies to epithelium-specific components of the human milk fat globule membrane: Production and reaction with cells in culture. *Int. J. Cancer* **28,** 17.

THOMSON, C.H., JONES, R.H., WHITEHEAD, S.L. & McKENZIE, I.F.C. (1983). A human breast tissue-associated antigen detected by a monoclonal antibody. *J. Natl Can. Inst.* **70,** 409.

WAALKES, P.T., GEHRKE, C.W., TORMEY, D.C. & 4 others. (1978). Biologic markers in breast carcinoma. IV. Serum fucose-protein ratio. Comparisons with carcinoembryonic antigen and human chorionic gonadtrophic. *Cancer* **41,** 1871.

WANG, D.Y., KNYBA, R.E.W., BULBROOK, R.D., MILLIS, R.R. & HAYWARD, J. (1984). Serum carcinoembryonic antigen in the diagnosis and prognosis of women with breast cancer. *Eur. J. Clin. Oncol.* **20,** 25.

WILKINSON, M.J.S., HOWELL, A., HARRIS, M., TAYLOR-PAPADIMITRIOU, J., SWINDELL, R. & SELLWOOD, R.A. (1984). The prognostic significance of two epithelial membrance antigens expressed by human mammary carcinomas. *Int. J. Cancer* **33,** 299.